# Admission hypo- or hyperthermia and survival after trauma in civilian and military environments

**DOI:** 10.1186/1865-1380-4-35

**Published:** 2011-06-23

**Authors:** Charles E Wade, José Salinas, Brian J Eastridge, John G McManus, John B Holcomb

**Affiliations:** 1 US Army Institute of Surgical Research, Fort Sam Houston, TX, USA; 2 Center for Translational Injury Research & Department of Surgery, The University of Texas Medical School at Houston, 6431 Fannin, MSB 5.204, Houston, TX, USA

## Abstract

**Background:**

In the care of patients with traumatic injuries, focus is placed on hypothermia secondary to its deleterious impact on the coagulation cascade. However, there is scant information on the mortality effect of hyperthermia.

**Study objectives:**

We hypothesized that both hypothermia and hyperthermia are associated with decreased survival in patients with traumatic injuries. Furthermore, we hypothesized that in the military setting, the incidence of hyperthermia would be greater compared to the civilian environment and thus contributing to an increase in mortality.

**Methods:**

Registries compared were the National Trauma Data Bank (NTDB), three civilian Level I trauma centers, and military combat support hospitals. The NTDB was used as a reference to define hypothermia and hyperthermia based upon survival. Admission temperature and outcome were known for 4,093 civilian and 4,394 military records.

**Results:**

Hypothermia was defined as < 36°C and hyperthermia > 38°C as mortality increased outside this range. The overall mortality rates were 3.5% for civilians and 2.5% for military (*p *< 0.05). Of civilians, 9.3% (382) were hypothermic and 2.2% (92) hyperthermic. The incidence of hypothermia in the military patients was 6.0% (263) and for hyperthermia the incidence was 7.4% (327). Irrespective of group, patients with hypothermia or hyperthermia had an increased mortality compared to those with normal temperatures, ([for civilian:military ] hypothermia 12%:11%; normal 2%:2%; hyperthermia 14%:4%).

**Conclusion:**

Care of the victim with traumatic injuries emphasizes avoidance of hypothermia; however, hyperthermia is also detrimental. The presence of hypothermia or hyperthermia should be considered in the initial treatment of the patient with traumatic injuries.

## Introduction

Body temperature has been identified as an essential element in the assessment of the patient requiring critical care. In the care of both civilian and military patients with traumatic injuries, focus has been placed on hypothermia upon patient admission to the hospital [1-13]. In these patients, hypothermia is associated with increases in coagulopathy, hemorrhage, multiple organ failure, length of hospital stay, and mortality [4,6-11,13]. Correction of hypothermia in the period of initial treatment is vigorously advocated and is associated with improved outcomes [14].

The presence of hyperthermia in the injured patient has received less attention than that of hypothermia. Malone et al. noted an increase in mortality in trauma patients if they had either hypothermia (< 36°C) or hyperthermia (> 38°C) [15]. Martin et al., in an analysis of the National Trauma Data Bank (NTDB), focused on hypothermia, and demonstrated a threefold increase in mortality in temperatures above 38°C [9]. At present, there are no guidelines on the initial treatment of the trauma patient admitted with hyperthermia after injury.

Martin et al. focused on trauma patients with hypothermia [9]. However, review of their NTDB data suggested that hyperthermia could also be associated with increased mortality. Hyperthermia may occur as a result of infection, high ambient temperatures, or increased physical effort. In elevated ambient temperature environments, the risk of primary hyperthermia can be exacerbated by heavy work or exercise. Present US military conflicts in Iraq and Afghanistan are occurring in countries with persistently high environmental temperatures. We hypothesized that in the present military conflicts, the incidence of hyperthermia in trauma patients upon admission would be greater than in a civilian setting. Further, the increase in the frequency of hyperthermia was expected to be associated with an increase in mortality and, therefore, correction of body temperature should be considered.

## Materials and methods

### Study design and setting

The study was approved by the Brooke Army Medical Center Institutional Review Board. Data were obtained from established registries. For the determination of the normal temperature range, the NTDB was queried. We used data from the San Antonio regional trauma centers, where the average annual ambient temperature is 20°C. Three Level I trauma centers serve this area of southwest Texas. The second data set was the NTDB registry, which contains comprehensive and demographic information extracted from patient records by trained registrars at each trauma center. All centers use a uniform customized software package (Collector Trauma Registry, Digital Innovation, Forest Hill, MD). The third data set was the Joint Theater Trauma Registry (JTTR), which consists of data on all US military trauma casualties from Iraq and Afghanistan, where the average annual temperature is 22°C. This data was extracted from medical records by trained registrars using software specifically designed for the military.

### Patient populations

To be included, patients had to have their body temperature recorded upon admission to the emergency department, and the hospital outcome had to be known. The outcome variable of interest was mortality, defined as death prior to hospital discharge. Data for both civilian and military groups were obtained from the period of July 2003 to January 2009. For the civilian group, the patients admitted to San Antonio Level I trauma centers were included. In the military group, patients were admitted to a combat support hospital (CSH). These facilities are capable of providing definitive surgical care and intensive care support. The resources available in the present US conflicts in Iraq and Afghanistan are often similar to those at a civilian Level I trauma center. Following stabilization, if necessary, patients were first transported to Germany, then onto the US. Military patients often reached a hospital in the US within 3 to 4 days after injury. Military patients were tracked though this system to determine final outcomes.

### Identification of groups

**Figure 1:**
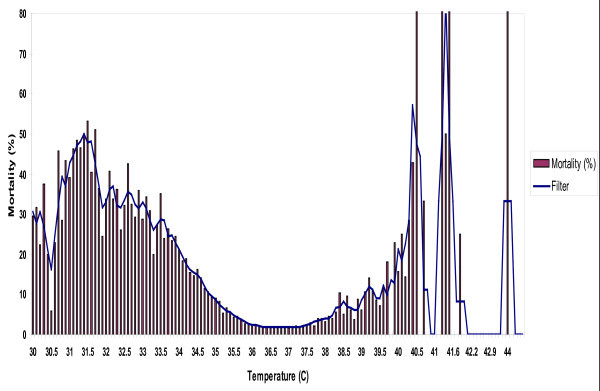
**Percent mortality at various body temperatures in patients with traumatic injuries as determined from the NTDB, *n *= 621,081**.

Patients were defined as either hypothermic or hyperthermic, based upon the body temperature recorded at admission to the emergency department. The definitions of hypothermia and hyperthermia were based on analysis of the relationship of body temperature to mortality data from the NTDB (Figure 1). Summary NTDB data were plotted and filtered by using window average filters to decimate peaks in the resulting data. Inflection points in the filtered data set were determined by calculating the data slope for each point in the filtered data set. Average, median, or average line slope estimation filters were used to generate a filtered slope set. Filters were executed by using a moving window across values in the resulting data set to generate the filtered results. Points of inflection in the final filtered data set were determined by the point at which the filtered slope values maintained a positive slope and did not cross the zero line.

### Analysis

Data were analyzed by using SAS version 8.1 (SAS Institute Inc., Cary, NC). For categorical variables, differences between groups were compared by using the chi-square test. For continuous variables, differences between groups were determined by analysis of variance following log transformation of the data to adjust for data heterogeneity. Post-hoc analysis to determine differences between group means used a Newman-Keuls test. Significance was set at *p *< 0.05. Values in the text are means ± standard deviation (SD).

## Results

NTDB data on 621,081 patients were available for analysis. Significant increases in mortality were noted in this patient set when body temperatures were < 36°C or > 38°C (Figure 1). Thus, deviations beyond these temperatures were used to define hypothermia and hyperthermia, respectively.

There were 4,093 civilian and 4,394 military patients with body temperatures recorded upon admission to the emergency department in whom outcomes were known. Of the civilian patients with known outcomes, 9.3% (382) were hypothermic and 2.2% (92) hyperthermic. The incidence of hypothermia in the military patients in which outcome was known was 6.0% (263) and for hyperthermia 7.4% (327). There were significant differences in the incidence of hypothermia and hyperthermia in the military population compared to the civilian population. The overall mortality rates were 3.5% for civilians and 2.4% for military (*p *< 0.05). Irrespective of group, patients with hypothermia or hyperthermia had an increased mortality compared to those with normal temperatures (36° to 38°C) (Table 1). There were no differences in mortality rates between the civilian and military groups for hypothermia and normal temperature groups. The increase in mortality in patients with hyperthermia was greater in the civilian patients than the military.

**Table 1:** Percent mortality in civilian and military patients

Study population	Hypothermia (< 36°C)	Normal (36°-38°C)	Hypothermia (> 38°C)
Civilian	12.3% (47/382)*	2.3% (84/3,619)	14.1% (13/92)*
Military	11.0% (29/263)*	1.7% (66/3,804)	3.7% (12/327)*^+^

*Significantly different (*p *< 0.05) from normal.

^+^Significantly different (*p *< 0.05) from civilian.

For the civilian population, there were significant mortality differences between the patients within the normal body temperature range and those with hypothermia and hyperthermia (Table 2). Those patients with hypothermia and hyperthermia had significant increases in indices of injury, more ventilator days, and greater lengths of stay in the intensive care unit (ICU) and in the hospital. Of note, there were no differences in physiological indices such as systolic pressure and revised trauma score (RTS), suggesting that differences in body temperature may be a delineating physiological factor in assessing civilian patient status on presentation.

**Table 2:** Demographic characteristics of the civilian population with known outcomes

Variable	**Hypothermia** **(< 36°C)**	**Normal** (36° -38°C)	**Hyperthermia** **(> 38°C)**
Body temperature (°C)	35.4 ± 0.91*	36.8 ± 0.52	38.6 ± 0.62*
Age (years)	41 ± 22.1	35 ± 19.2	34 ± 22.1
Systolic blood pressure (mmHg)	127 ± 33.0	134 ± 29.5	135 ± 30.8
Injury severity score (ISS)	14 ± 14.4*	8 ± 9.0	14 ± 13.0*
RTS	11 ± 2.4	12 ± 1.2	11 ± 1.5
TRISS	0.84 ± 0.282*	0.96 ± 0.122	0.87 ± 0.234*
Intensive care unit length of stay (days)	4 ± 8.9*	1 ± 5.6	3 ± 5.1*
Ventilator days	2 ± 7.8*	1 ± 4.6	2 ± 3.6*
Hospital length of stay (days)	9 ± 21.7*	5 ± 10.1	8 ± 9.8*

Values are mean ± SD.

*Significantly different from the 36°-38°C group.

In military patients, there were indices of increased severity of injury in patients with hypothermia and hyperthermia (Table 3). The injury severity score (ISS) was increased, and the trauma revised ISS (TRISS) was reduced in these groups. The RTS was not different between groups, although with hypothermia, a significantly lower mean systolic blood pressure was noted. Patients with hypothermia or hyperthermia spent a greater number of days on ventilation and in the ICU. There was no significant difference in the length of hospital stay. On the whole, military patients, while more severely injured and younger, showed similar mortality differences as those seen for civilian patients in the effects of hypothermia and hyperthermia.

**Table 3:** Demographic characteristics of the military population with known outcomes.

Variable	**Hypothermia** **(< 36°C)**	**Normal** (36°-38°C)	**Hyperthermia** **(> 38°C)**
Body temperature (°C)	35.2 ± 1.07*	36.9 ± 0.46	38.6 ± 0.41*
Age (years)	26 ± 6.5	26 ± 6.7	26 ± 6.0
Systolic blood pressure (mmHg)	130 ± 45.7	131 ± 17.5	126 ± 22.7*
Injury severity score (ISS)	21 ± 15.6*	13 ± 11.1	21 ± 13.0*
RTS	6 ± 2.7	7 ± 2.1	6 ± 2.1
TRISS	0.80 ± 0.282*	0.95 ± 0.147	0.83 ± 0.255*
Intensive care unit length of stay (days)	1 ± 2.0*	0.8 ± 2.1	3 ± 4.4*
Ventilator days	1 ± 1.6*	0.5 ± 1.5	2 ± 2.8*
Hospital length of stay(days)	2 ± 5.6	4 ± 26.6	6 ± 15.5

Values are mean ± SD.

*Significantly different from the normal temperature group, 36°-38°C group.

In the military population, the Barell index for traumatic brain injury (TBI) was recorded and the anatomical injury score (AIS) for brain trauma was coded. The incidence of moderate or severe brain injury with the Barell index in patients was 55% in the hypothermia group, 36% in the normal group, and 49% in the hyperthermia group. With an AIS > 2, there was a 53% rate of brain injury for the hypothermia group, 35% for the normal group, and 56% for the hyperthermia group. For both measures of brain injury, the rates were increased in patients with hypothermia and hyperthermia compared to normal. There were no differences between hypothermia and hyperthermia in the incidence of TBI.

## Discussion

Hypothermia is a well-defined contributor to poor outcomes in patients with traumatic injuries [1-13]. The presence of hypothermia is associated with coagulopathy and increased blood transfusion requirements as well as mortality. The influence of hyperthermia on trauma mortality has not often been addressed. In an evaluation of using the systemic inflammatory response syndrome (SIRS) score to assess outcomes in patients with traumatic injuries, others noted that altered temperature (either hypothermia or hyperthermia) was a significant predictor of outcome [15-18]. In the present study, we referenced the NTDB data to determine the range of normal temperature, based on when the rate of mortality increased. Although the NTDB and other registries have numerous limitations, these databases allow access to information of large numbers of patients to describe the population of interest. A range for normal values was found to be 36° to 38°C using data from over 600,000 patients. The hypothermic threshold was identical to that in earlier studies of this data set [9,13]. These studies of hypothermia suggested increases in mortality below body temperatures of 36°C; however, the hyperthermic threshold was not previously defined. In the present study of military and civilian populations, the impact of hypothermia or hyperthermia was associated with similar increases in mortality, compared to patients with temperatures in the range of 36° to 38°C.

There were differences in the incidence rates of hypothermia and hyperthermia between the military and the civilian populations. The greater incidence of hyperthermia in the military population and the reduction in hypothermia may be the result of the ambient environment, differences in clothing worn, and the patients' activities at the time of injury. The average ambient temperature over the year in San Antonio where the civilian population was evaluated was 20°C, with an average high of 27°C and a low of 14°C. In Iraq, where 95% of the military population was studied, the average annual environmental temperature was 22°C, with the average high and low temperature of 30° and 15°C, respectively (UN World Meteorological Organization). Average temperatures did not appear significantly different between the two study sites. In theater, soldiers wear personnel protective equipment and heavy clothing, which limit heat loss. Furthermore, there may be significant differences in other factors; for example, at the time of injury, soldiers are often on foot and engaged in physical activity in contrast to civilians who may be riding in air-conditioned automobiles. The greater occurrence of hyperthermia may also be the result of increased incidence of brain injuries [19]. However, within the military population, the incidence of severe traumatic injury, while greater than in patients with normal temperatures, was not different between patients with hypothermia or hyperthermia. The increase in ambient temperature, protective clothing worn, types of injuries, and the work activities experienced by the military population may have contributed to the increased incidence of hyperthermia and the reduced incidence of hypothermia in contrast to the incidence observed in the civilian population.

A greater probability of mortality in the civilian population was associated with hyperthermia and may be the result of a number of factors. There are distinct differences in age between civilian and military casualties [20]. Civilian casualties are generally older and, on average, are affected by comorbid disease factors. The cause of injury in civilian patients is often blunt in contrast to penetrating, with or without explosion, in military casualties. In addition, the extent of injury may have been different between the populations. Though a group difference in outcome between the military and civilian subjects was observed, the increase in mortality with alterations in body temperature was noted in both populations.

On admission, neither population showed a difference between temperature groups in physiological indices (such as systolic blood pressure and RTS), which are normally used acutely in evaluating the status of a patient. Thus, the differences in body temperature may be a delineating physiological factor in assessing patient status and subsequent outcome, as previously suggested by others [9,13,16,17].

## Limitations

This is a retrospective study with the inherent limitations associated with this methodology. Registry data were used that are not the primary source, which may include transcription errors. That said, the size of the patient population studies was an attempt to minimize these errors. Finally, we only used patients in which complete data were available and outcomes were known. This may have led to some selection bias.

## Conclusions

Deviations in body temperature above or below the range of 36° to 38°C in the patients admitted to the hospital with traumatic injuries are indicative of a poor outcome and, therefore, should be addressed as soon as possible. This indication is similar to the findings observed in patients with an increased SIRS due to increased body temperature upon admission [15-18]. In the care of the patient with traumatic injuries, emphasis has been placed on avoiding hypothermia; however, hyperthermia is similarly detrimental. Hyperthermia may be the result of ambient temperature, clothing worn, or work environment as in the present military conflicts. Although a greater incidence rate of hyperthermia was observed in the military group compared to the civilian group, a greater mortality rate was not apparent. The presence of hypothermia or hyperthermia should be considered in the initial treatment of the patient with traumatic injuries and corrected to the normal range of 36° to 38°C.

## Competing interests

The authors declare that they have no competing interests.

## Authors' contributions

CEW and JS conceived and designed the study. CEW and BJE participated in the collection of data. CEW, JS, and BJE preformed the statistical analysis and interpreted the data. CEW, JS, BJE, JGM, and JBH drafted the manuscript and contributed to critical revisions. All authors have read and approved the final manuscript.
